# Adhesion-guided CEI enables stable 4.6 V LiCoO_2_ at 45°C

**DOI:** 10.1093/nsr/nwaf571

**Published:** 2025-12-23

**Authors:** Zhikang Deng, Peng Liang, Ai-Min Li, Hongjie Dai

**Affiliations:** Department of Chemistry, The University of Hong Kong, China; Material Innovation Institute for Life Sciences and Energy (MILES), HKU-SIRI, China; JC STEM Lab of Nanoscience and Nanomedicine, Department of Chemistry and School of Biomedical Sciences, The University of Hong Kong, China; Department of Chemistry, The University of Hong Kong, China; Material Innovation Institute for Life Sciences and Energy (MILES), HKU-SIRI, China; JC STEM Lab of Nanoscience and Nanomedicine, Department of Chemistry and School of Biomedical Sciences, The University of Hong Kong, China; Department of Chemistry, The University of Hong Kong, China; Material Innovation Institute for Life Sciences and Energy (MILES), HKU-SIRI, China; Department of Chemistry, The University of Hong Kong, China; Material Innovation Institute for Life Sciences and Energy (MILES), HKU-SIRI, China; JC STEM Lab of Nanoscience and Nanomedicine, Department of Chemistry and School of Biomedical Sciences, The University of Hong Kong, China; Department of Chemistry, Stanford University, USA

Electrochemical interfaces are the fundamental stages where ion transport, charge transfer and chemical transformation occur, ultimately dictating the stability and performance of rechargeable batteries [1]. In high-voltage lithium-ion batteries, the cathode–electrolyte interphase (CEI) plays a decisive role in sustaining long-term cell operation. While a compact CEI is generally sufficient under room-temperature cycling, challenges become far more severe at elevated potentials and temperatures. When cycled above 4.5 V and at increased temperature, LiCoO_2_ (LCO) undergoes accelerated electrolyte oxidation, lattice oxygen release and interphase breakdown [2,3]. In such harsh conditions, parasitic reactions are intensified, and certain CEI components may also be dissolved, resulting in rapid cell degradation. Therefore, the CEI must not only be compact but also exhibit high thermal stability and strong adhesion to the LCO particles.

**Figure 1.**
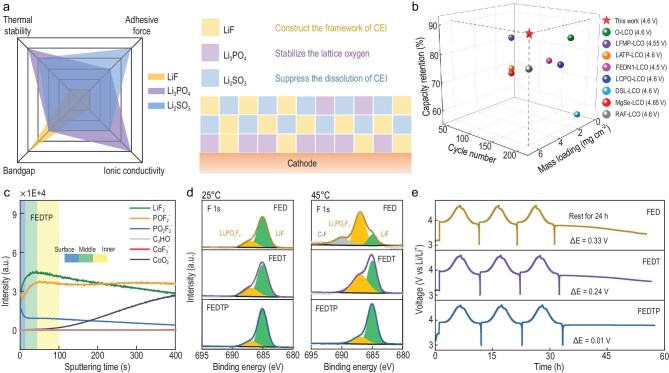
(a) Radar charts of three inorganic components and a schematic illustration of CEI design. The bandgap and ionic conductivity data are collected from the previous research and material database. (b) Comparison of the LCO electrochemical performance under elevated temperature conditions. (c) Depth profiles of various secondary ion fragments during the ToF-SIMS measurement for LCO cathodes with FEDTP electrolytes after 200 cycles at 45°C. (d) F 1s XPS spectra of LCO cathodes with various electrolytes after 200 cycles at 25 or 45°C. (e) Time–voltage curves of LCO cathodes with various electrolytes under 0.2 C current density at 45°C. Reproduced from Chen *et al*. [4] with permission.

To meet these dual requirements, Chen *et al.* proposed using lithium phosphate (Li_3_PO_4_) for its superior thermal stability and moderate ionic conductivity, in combination with lithium sulfite (Li_2_SO_3_) for its strong adhesion. Both species were identified through density functional theory (DFT) calculations capable of addressing the challenges of high-voltage cell operation under thermal stress (Fig. 1a) [4]. By leveraging triethyl phosphate (TEP) and 1,3-propane sultone (PS) in the electrolyte, they achieved the *in*  *situ* construction of an inorganic-rich CEI composed of Li_3_PO_4_, LiF and Li_2_SO_3_. This adhesion-guided architecture ensures that the CEI remains intact, mechanically robust and chemically stable under high-voltage and elevated-temperature conditions.

At 4.6 V and 45°C, Li||LCO cells with the designed electrolyte (denoted as FEDTP) exhibit excellent cycling stability (Fig. 1b), delivering an initial capacity of ∼190 mAh g^−1^ and retaining 81.9% capacity after 500 cycles with a Coulombic efficiency of ∼99.9%. This approach is also proved to be effective in the 1 Ah LCO||graphite pouch cells, where FEDTP significantly reduces capacity fade and voltage decay at elevated temperatures. To understand the origin of this improvement, both experimental and computational analyses were conducted on the solvation structure of the developed electrolytes. Spectroscopy and molecular dynamics simulations revealed that TEP and PS regulate the Li⁺ solvation environment, lowering the desolvation barrier and directing preferential oxidation toward the formation of Li_3_PO_4_ and Li_2_SO_3_ at the cathode surface. Depth profiling and time-of-flight secondary ion mass spectroscopy (ToF-SIMS) analyses confirmed the formation of a trilayer CEI with an inner layer of Li_3_PO_4_, middle layer of LiF and outer layer of Li_2_SO_3_ (Fig. 1c).

Morphological and mechanical characterizations further highlighted the robustness of this architecture, demonstrating that the CEI is uniform and thin, with enhanced mechanical strength compared to conventional interphases. The improved thermal stability of the trilayer CEI is validated by X-ray photoelectron spectroscopy (XPS) measurements, which show suppressed surface degradation and a stable chemical composition at elevated temperatures (Fig. 1d). Electrochemical profiles further confirmed that this CEI resists dissolution, as reflected by the sustained voltage curves under prolonged cycles (Fig. 1e). Collectively, these results demonstrate that the adhesion and cohesion in CEI design, which have long been overlooked, are decisive parameters for maintaining superior interfacial stability under harsh conditions.

In summary, this work demonstrates that beyond regulating the chemical composition and spatial distribution of the CEI, synergistic control over binding interactions between CEI components and cathode particles can effectively extend the operational boundary for high-voltage cathodes. Looking forward, this concept is not only transferable to other cathode materials but also offers a promising strategy for stabilizing post-lithium systems, particularly sodium-ion batteries, where electrolyte–electrode interphase dissolution remains a critical issue [5–8].
